# Bis(4,4′-bipyridin-1-ium) *cis*-bis­(1,2-di­cyano-2-sulfido­ethene-1-sulfinato-κ^2^*S*,*S*′)platinate(2−)

**DOI:** 10.1107/S2414314625003839

**Published:** 2025-05-23

**Authors:** Claire E. Welton, Eric W. Reinheimer, Bradley W. Smucker

**Affiliations:** a 900 N Grand Avenue, Suite 61651, Austin College, Sherman, TX 75090, USA; b Rigaku Oxford Diffraction, 9009 New Trails Dr., The Woodlands, TX 77381, USA; Benemérita Universidad Autónoma de Puebla, México

**Keywords:** crystal structure, sulfinate, platinum, salt, pyridinium

## Abstract

Crystals of the sulfinate-containing platinate salt, (C_10_H_9_N_2_)_2_[Pt(C_4_N_2_O_2_S_2_)_2_], were obtained from a solution of [Pt(4,4′-bpy)_2_(mnt)] after a long period with minimal light (4,4′-bpy is 4,4′-bi­pyridine and mnt is maleo­nitrile­dithiol­ate). In the crystals, the cations associate via C—H⋯O hydrogen bonds with hydrogen bonded (—H⋯) chains of pyridinium anions. The *cis*-geometry of the sulfinate around the square-planar platinum atom, coupled with a torsion of one of the rings of the pyridinium, engenders multiple hydrogen bonds between the sulfinate oxygen atoms and the pyridinium hydrogen atoms.

## Abstract 2

The crystal structure of the title sulfinate-containing platinate salt features cationic chains composed of 4,4′-bipyridin-1-ium surrounding slipped-stacked anions of platinum doubly chelated by a sulfinato ligand.

## Graphical abstract

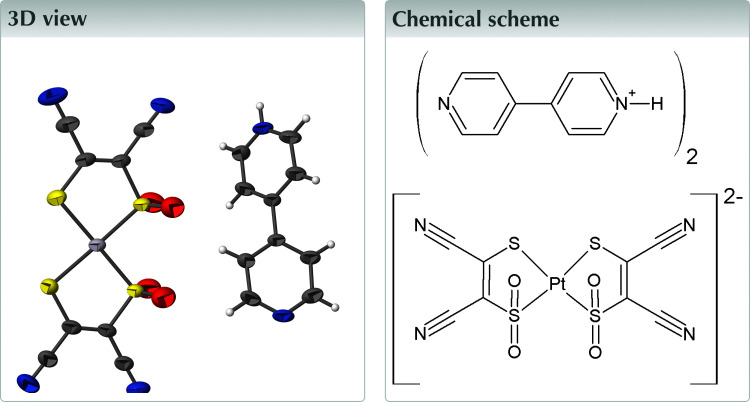

## Structure description

**Figure 1:**
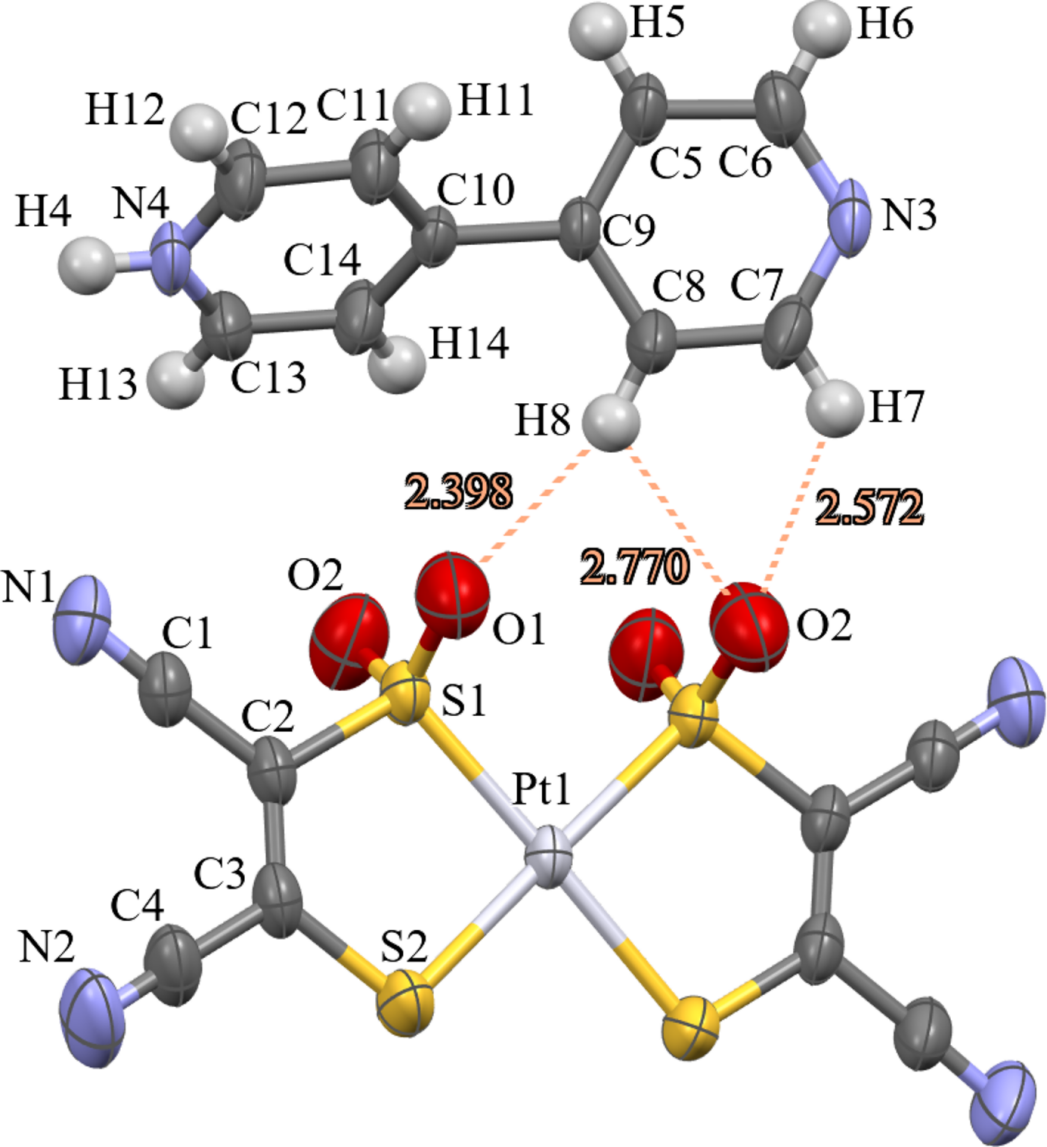
Ellipsoid representation (50% probability) of the title compound showing O⋯H distances for hydrogen bonds between O1 and H8 and O2(−*x* + 1, *y*, −*z* + 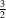) and H8 and H7.

The asymmetric unit of the title compound contains one-half of the platinum bis-sulfinate moiety with the platinum residing on an inversion centre and a single pyridinium cation. The square-planar platinate anions have the sulfinate moieties in a *cis* arrangement around the platinum atom (Fig. 1 ▸). The Pt—S distances between the S2 thiol­ate and the S1 sulfinate are 2.3120 (13) and 2.2554 (12) Å, respectively. The S=O bond distances [S1=O1 = 1.448 (4) and S1=O2 = 1.439 (4) Å] in this structure match the S=O distances of 1.470 (4) and 1.444 (4) Å observed in platinum(II) sulfinato-thiol­ato complexes (Ishii *et al.*, 2007 ▸). The S—O single-bond distances in sulfenato ligands typically are around 0.1 Å longer at 1.55 Å (Buonomo *et al.*, 1995 ▸).

**Figure 2:**
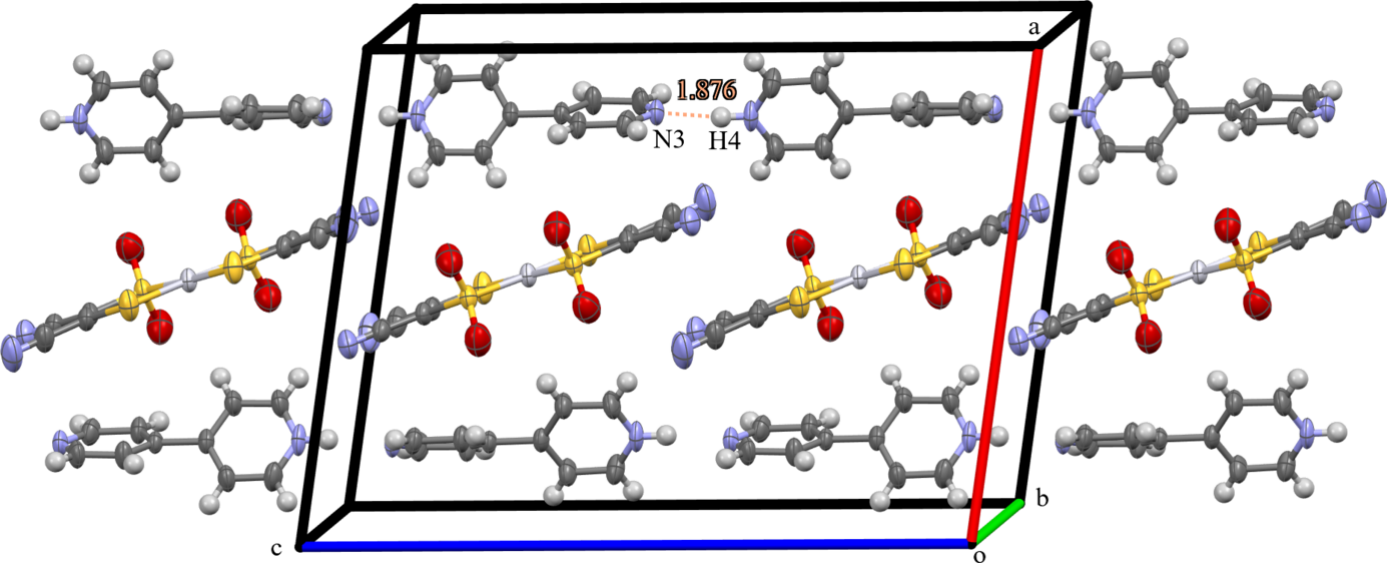
Ellipsoid representation (50% probability) of the packing of the title compound with pyridinium chains connected by hydrogen bonds between N3 of one pyridinium and the H4 of an adjacent pyridinium. The H-bond distance between N3(−*x* + 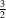, *y* + 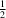, −*z* + 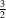) and H4(−*x* + 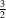, −*y* + 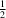, −*z* + 1) is shown. Some symmetry-related cell contents were removed for clarity.

The pyridinium cations form hydrogen-bonded one-dimensional chains along the *c*-axis direction, which surround the platinate anions. These anions are slipped-stacked so as to fit between the pyridinium chains (Fig. 2 ▸). This type of packing of the anions is observed in a [Pt(mnt)_2_]^−^ salt with a planar 4-amino­pyridinium monocation (Pei *et al.*, 2012 ▸), but when [Pt(mnt)_2_]^2−^ crystallizes with a planar dicationic [4,4′-H_2_bpy]^2+^ it forms *ABAB* stacks of alternating cations and anions (Crawford *et al.*, 2004 ▸). The sulfinate moieties alternate their positions along the slipped stacking of the anions, which corresponds to a rotation between the two rings of the pyridinium cation [torsion angle C8—C9—C10—C11 of 130.8 (5)°], that positions the H8 and H7 hydrogen atoms for H-bonding with the O1 and O2(−*x* + 1, *y*, −*z* + 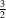) atoms of the sulfinates (Table 1 ▸ and Fig. 1 ▸).

## Synthesis and crystallization

A methanol/water (1:1) solution containing [Pt(4,4′-bpy)_2_(mnt)] (Smith *et al.* 2019 ▸; 4,4′-bpy is 4,4′-bi­pyridine and mnt is maleo­nitrile­dithiol­ate) was combined with excess 4,4′-bpy due to the observed exchangeability of the 4,4′-bpy ligand. This solution was layered with THF in a thin tube. Small orange prisms were harvested from the bottom of the tube after a long period with minimal light. This conversion of a di­thiol­ate ligand to its monosulfinate derivative has been achieved through multiple modes including: photooxidation in the presence of water (Connick & Gray, 1997 ▸), chemical oxidation (Sugimoto *et al.*, 2000 ▸; Ishii *et al.*, 2007 ▸), or after a prolonged period in minimal light (Stace *et al.*, 2016 ▸). Protons for the pyridinium likely originated from the solvents.

## Refinement

Crystal data, data collection, and refinement details are summarized in Table 2 ▸.

## Supplementary Material

Crystal structure: contains datablock(s) I. DOI: 10.1107/S2414314625003839/bh4095sup1.cif

Structure factors: contains datablock(s) I. DOI: 10.1107/S2414314625003839/bh4095Isup2.hkl

CCDC reference: 2447480

Additional supporting information:  crystallographic information; 3D view; checkCIF report

## Figures and Tables

**Table 1:** Hydrogen-bond geometry (Å, °)

*D*—H⋯*A*	*D*—H	H⋯*A*	*D*⋯*A*	*D*—H⋯*A*
N4—H4⋯N3^i^	0.86	1.88	2.730 (5)	171
C7—H7⋯N2^ii^	0.93	2.50	3.363 (7)	155
C7—H7⋯O2^iii^	0.93	2.57	3.197 (7)	125
C8—H8⋯O1	0.93	2.40	3.301 (6)	164
C8—H8⋯O2^iii^	0.93	2.77	3.287 (7)	116

Symmetry codes: (i) 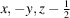; (ii) 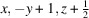; (iii) 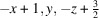.

**Table 2:** Experimental details

Crystal data	
Chemical formula	(C_10_H_9_N_2_)_2_[Pt(C_4_N_2_O_2_S_2_)_2_]
*M*_r_	853.83
Crystal system, space group	Monoclinic, *C*2/*c*
Temperature (K)	293
*a*, *b*, *c* (Å)	14.5669 (8), 10.8981 (6), 19.4595 (9)
β (°)	98.782 (5)
*V* (Å^3^)	3053.0 (3)
*Z*	4
Radiation type	Mo *K*α
μ (mm^−1^)	4.92
Crystal size (mm)	0.09 × 0.07 × 0.03

Data collection	
Diffractometer	XtaLAB Mini II
Absorption correction	Analytical (*CrysAlis PRO*; Rigaku OD, 2024 ▸)
*T*_min_, *T*_max_	0.712, 0.851
No. of measured, independent and observed [*I* > 2σ(*I*)] reflections	21005, 3124, 2503
*R*_int_	0.042
(sin θ/λ)_max_ (Å^−1^)	0.625

Refinement	
*R*[*F*^2^ > 2σ(*F*^2^)], *wR*(*F*^2^), *S*	0.031, 0.066, 1.04
No. of reflections	3124
No. of parameters	204
H-atom treatment	H-atom parameters constrained
Δρ_max_, Δρ_min_ (e Å^−3^)	0.71, −0.52

Computer programs: *CrysAlis PRO* (Rigaku OD, 2024 ▸), *SHELXT2018/2* (Sheldrick, 2015*a* ▸), *SHELXL2014/7* (Sheldrick, 2015*b* ▸), *Mercury* (Macrae *et al.*, 2020 ▸), *OLEX2* (Dolomanov *et al.*, 2009 ▸) and *publCIF* (Westrip, 2010 ▸).

## full crystallographic data

### Bis(4,4'-bipyridin-1-ium) cis-bis(1,2-dicyano-2-sulfidoethene-1-sulfinato-κ2S,S')platinate(2-) Crystal data

**Table:**

(C_10_H_9_N_2_)_2_[Pt(C_4_N_2_O_2_S_2_)_2_]	*F*(000) = 1664
*M**_r_* = 853.83	*D*_x_ = 1.858 Mg m^−^^3^
Monoclinic, *C*2/*c*	Mo *K*α radiation, λ = 0.71073 Å
*a* = 14.5669 (8) Å	Cell parameters from 2218 reflections
*b* = 10.8981 (6) Å	θ = 2.4–25.0°
*c* = 19.4595 (9) Å	µ = 4.92 mm^−^^1^
β = 98.782 (5)°	*T* = 293 K
*V* = 3053.0 (3) Å^3^	Irregular, orange
*Z* = 4	0.09 × 0.07 × 0.03 mm

### Bis(4,4'-bipyridin-1-ium) cis-bis(1,2-dicyano-2-sulfidoethene-1-sulfinato-κ2S,S')platinate(2-) Data collection

**Table:**

XtaLAB Mini II diffractometer	3124 independent reflections
Radiation source: fine-focus sealed X-ray tube, Rigaku (Mo) X-ray Source	2503 reflections with *I* > 2σ(*I*)
Graphite monochromator	*R*_int_ = 0.042
Detector resolution: 10.0000 pixels mm^-1^	θ_max_ = 26.4°, θ_min_ = 2.5°
ω scans	*h* = −18→18
Absorption correction: analytical (CrysAlisPro; Rigaku OD, 2024)	*k* = −13→13
*T*_min_ = 0.712, *T*_max_ = 0.851	*l* = −22→24
21005 measured reflections	

### Bis(4,4'-bipyridin-1-ium) cis-bis(1,2-dicyano-2-sulfidoethene-1-sulfinato-κ2S,S')platinate(2-) Refinement

**Table:**

Refinement on *F*^2^	Primary atom site location: dual
Least-squares matrix: full	Secondary atom site location: difference Fourier map
*R*[*F*^2^ > 2σ(*F*^2^)] = 0.031	Hydrogen site location: inferred from neighbouring sites
*wR*(*F*^2^) = 0.066	H-atom parameters constrained
*S* = 1.04	*w* = 1/[σ^2^(*F*_o_^2^) + (0.0297*P*)^2^ + 5.2814*P*] where *P* = (*F*_o_^2^ + 2*F*_c_^2^)/3
3124 reflections	(Δ/σ)_max_ = 0.001
204 parameters	Δρ_max_ = 0.71 e Å^−^^3^
0 restraints	Δρ_min_ = −0.52 e Å^−^^3^

### Bis(4,4'-bipyridin-1-ium) cis-bis(1,2-dicyano-2-sulfidoethene-1-sulfinato-κ2S,S')platinate(2-) Special details

**Table:**

Refinement. H atoms bound to C and N atoms were positioned geometrically (C—H = 0.93 Å and N—H = 0.86 Å) and constrained to ride on the parent atom. *U*_iso_(H) values were fixed at multiples of *U*_eq_(C) [1.2 for C(H) and N(H) groups].

### Bis(4,4'-bipyridin-1-ium) cis-bis(1,2-dicyano-2-sulfidoethene-1-sulfinato-κ2S,S')platinate(2-) Fractional atomic coordinates and isotropic or equivalent isotropic displacement parameters (Å^2^)

**Table:**

	*x*	*y*	*z*	*U*_iso_*/*U*_eq_	
Pt1	0.5000	0.54391 (3)	0.7500	0.03897 (10)	
S1	0.53256 (8)	0.39852 (12)	0.67484 (5)	0.0419 (3)	
S2	0.53745 (10)	0.69516 (13)	0.67571 (6)	0.0578 (4)	
O1	0.6114 (3)	0.3230 (4)	0.7024 (2)	0.0851 (13)	
O2	0.4521 (3)	0.3318 (4)	0.6426 (2)	0.0870 (13)	
N1	0.6220 (3)	0.3574 (5)	0.5069 (2)	0.0660 (13)	
N2	0.6297 (4)	0.7379 (5)	0.5134 (2)	0.0909 (18)	
C1	0.5992 (3)	0.4160 (5)	0.5502 (2)	0.0463 (13)	
C2	0.5716 (3)	0.4857 (5)	0.6059 (2)	0.0422 (12)	
C3	0.5720 (3)	0.6082 (5)	0.6092 (2)	0.0463 (12)	
C4	0.6036 (4)	0.6797 (5)	0.5551 (3)	0.0607 (15)	
N3	0.6676 (3)	−0.0315 (4)	0.91351 (18)	0.0501 (10)	
N4	0.6763 (3)	0.0238 (4)	0.55456 (18)	0.0539 (11)	
H4	0.6777	0.0327	0.5108	0.065*	
C5	0.6851 (3)	−0.1265 (5)	0.8051 (2)	0.0490 (12)	
H5	0.6972	−0.1970	0.7811	0.059*	
C6	0.6820 (3)	−0.1305 (6)	0.8767 (2)	0.0530 (13)	
H6	0.6904	−0.2057	0.8994	0.064*	
C7	0.6538 (4)	0.0747 (5)	0.8800 (2)	0.0566 (15)	
H7	0.6444	0.1445	0.9055	0.068*	
C8	0.6526 (4)	0.0866 (5)	0.8087 (2)	0.0513 (13)	
H8	0.6403	0.1621	0.7870	0.062*	
C9	0.6698 (3)	−0.0150 (4)	0.7708 (2)	0.0371 (11)	
C10	0.6723 (3)	−0.0038 (4)	0.6942 (2)	0.0390 (11)	
C11	0.7447 (3)	−0.0520 (5)	0.6645 (2)	0.0512 (13)	
H11	0.7928	−0.0941	0.6915	0.061*	
C12	0.7449 (4)	−0.0366 (5)	0.5939 (2)	0.0578 (14)	
H12	0.7934	−0.0689	0.5735	0.069*	
C13	0.6066 (4)	0.0702 (5)	0.5822 (2)	0.0557 (14)	
H13	0.5595	0.1119	0.5539	0.067*	
C14	0.6023 (4)	0.0579 (5)	0.6520 (2)	0.0537 (13)	
H14	0.5526	0.0908	0.6706	0.064*	

### Bis(4,4'-bipyridin-1-ium) cis-bis(1,2-dicyano-2-sulfidoethene-1-sulfinato-κ2S,S')platinate(2-) Atomic displacement parameters (Å^2^)

**Table:**

	*U*^11^	*U*^22^	*U*^33^	*U*^12^	*U*^13^	*U*^23^
Pt1	0.04944 (16)	0.04359 (17)	0.02506 (13)	0.000	0.00946 (10)	0.000
S1	0.0532 (7)	0.0454 (7)	0.0290 (6)	−0.0023 (6)	0.0125 (5)	0.0011 (5)
S2	0.0908 (10)	0.0468 (8)	0.0387 (7)	−0.0084 (7)	0.0192 (7)	0.0017 (6)
O1	0.106 (3)	0.090 (3)	0.061 (2)	0.019 (3)	0.017 (2)	0.006 (2)
O2	0.096 (3)	0.098 (4)	0.070 (3)	−0.023 (3)	0.023 (2)	−0.016 (3)
N1	0.074 (3)	0.089 (4)	0.039 (2)	0.001 (3)	0.019 (2)	−0.009 (3)
N2	0.133 (5)	0.095 (4)	0.048 (3)	−0.041 (4)	0.024 (3)	0.010 (3)
C1	0.048 (3)	0.060 (4)	0.030 (2)	−0.005 (2)	0.005 (2)	0.002 (2)
C2	0.046 (3)	0.055 (4)	0.027 (2)	−0.001 (2)	0.008 (2)	0.006 (2)
C3	0.050 (3)	0.061 (4)	0.027 (2)	−0.004 (3)	0.006 (2)	0.006 (2)
C4	0.080 (4)	0.067 (4)	0.036 (3)	−0.020 (3)	0.010 (3)	0.003 (3)
N3	0.060 (2)	0.071 (3)	0.0202 (17)	0.005 (2)	0.0091 (17)	0.000 (2)
N4	0.086 (3)	0.058 (3)	0.0185 (17)	−0.007 (3)	0.010 (2)	0.0003 (19)
C5	0.066 (3)	0.057 (3)	0.024 (2)	−0.005 (3)	0.008 (2)	−0.004 (2)
C6	0.065 (3)	0.064 (4)	0.029 (2)	−0.004 (3)	0.003 (2)	0.007 (3)
C7	0.073 (4)	0.068 (4)	0.029 (2)	0.014 (3)	0.010 (2)	−0.013 (2)
C8	0.076 (4)	0.051 (3)	0.028 (2)	0.015 (3)	0.010 (2)	0.000 (2)
C9	0.045 (2)	0.045 (3)	0.021 (2)	−0.002 (2)	0.0061 (18)	−0.0001 (19)
C10	0.053 (3)	0.041 (3)	0.023 (2)	−0.004 (2)	0.007 (2)	−0.0033 (18)
C11	0.060 (3)	0.066 (4)	0.028 (2)	0.006 (3)	0.010 (2)	0.000 (3)
C12	0.070 (3)	0.077 (4)	0.030 (2)	0.005 (3)	0.019 (2)	−0.001 (3)
C13	0.080 (4)	0.057 (4)	0.029 (2)	0.012 (3)	0.004 (2)	0.004 (2)
C14	0.067 (3)	0.067 (4)	0.029 (2)	0.011 (3)	0.013 (2)	−0.004 (2)

### Bis(4,4'-bipyridin-1-ium) cis-bis(1,2-dicyano-2-sulfidoethene-1-sulfinato-κ2S,S')platinate(2-) Geometric parameters (Å, º)

**Table:**

Pt1—S1^i^	2.2554 (12)	C5—H5	0.9300
Pt1—S1	2.2554 (12)	C5—C6	1.402 (6)
Pt1—S2^i^	2.3120 (13)	C5—C9	1.387 (7)
Pt1—S2	2.3120 (13)	C6—H6	0.9300
S1—O1	1.448 (4)	C7—H7	0.9300
S1—O2	1.439 (4)	C7—C8	1.392 (6)
S1—C2	1.805 (5)	C8—H8	0.9300
S2—C3	1.740 (5)	C8—C9	1.375 (6)
N1—C1	1.147 (6)	C9—C10	1.501 (5)
N2—C4	1.139 (6)	C10—C11	1.382 (6)
C1—C2	1.431 (7)	C10—C14	1.383 (7)
C2—C3	1.337 (7)	C11—H11	0.9300
C3—C4	1.440 (7)	C11—C12	1.384 (6)
N3—C6	1.330 (6)	C12—H12	0.9300
N3—C7	1.328 (6)	C13—H13	0.9300
N4—H4	0.8600	C13—C14	1.376 (6)
N4—C12	1.335 (7)	C14—H14	0.9300
N4—C13	1.321 (6)		
			
S1^i^—Pt1—S1	90.74 (6)	N3—C6—C5	122.8 (5)
S1—Pt1—S2	90.13 (4)	N3—C6—H6	118.6
S1^i^—Pt1—S2	178.08 (5)	C5—C6—H6	118.6
S1^i^—Pt1—S2^i^	90.13 (4)	N3—C7—H7	118.4
S1—Pt1—S2^i^	178.08 (5)	N3—C7—C8	123.3 (5)
S2^i^—Pt1—S2	89.05 (7)	C8—C7—H7	118.4
O1—S1—Pt1	113.09 (17)	C7—C8—H8	120.5
O1—S1—C2	104.6 (2)	C9—C8—C7	119.0 (5)
O2—S1—Pt1	113.54 (18)	C9—C8—H8	120.5
O2—S1—C2	105.7 (2)	C5—C9—C10	121.5 (4)
C2—S1—Pt1	103.49 (17)	C8—C9—C5	118.4 (4)
C3—S2—Pt1	101.52 (18)	C8—C9—C10	120.1 (4)
N1—C1—C2	178.0 (6)	C11—C10—C9	121.4 (4)
C1—C2—S1	116.2 (4)	C11—C10—C14	118.5 (4)
C3—C2—S1	119.4 (4)	C14—C10—C9	120.1 (4)
C3—C2—C1	124.5 (4)	C10—C11—H11	120.4
C2—C3—S2	125.4 (4)	C10—C11—C12	119.1 (5)
C2—C3—C4	120.4 (5)	C12—C11—H11	120.4
C4—C3—S2	114.2 (4)	N4—C12—C11	120.9 (5)
N2—C4—C3	178.6 (6)	N4—C12—H12	119.5
C7—N3—C6	117.9 (4)	C11—C12—H12	119.5
C12—N4—H4	119.7	N4—C13—H13	119.4
C13—N4—H4	119.7	N4—C13—C14	121.2 (5)
C13—N4—C12	120.7 (4)	C14—C13—H13	119.4
C6—C5—H5	120.7	C10—C14—H14	120.2
C9—C5—H5	120.7	C13—C14—C10	119.5 (5)
C9—C5—C6	118.6 (5)	C13—C14—H14	120.2
			
Pt1—S1—C2—C1	178.9 (3)	C6—N3—C7—C8	0.7 (8)
Pt1—S1—C2—C3	−1.9 (4)	C6—C5—C9—C8	0.3 (7)
Pt1—S2—C3—C2	1.7 (5)	C6—C5—C9—C10	179.8 (4)
Pt1—S2—C3—C4	−177.6 (3)	C7—N3—C6—C5	1.5 (7)
S1—C2—C3—S2	0.1 (6)	C7—C8—C9—C5	1.7 (7)
S1—C2—C3—C4	179.4 (4)	C7—C8—C9—C10	−177.8 (5)
O1—S1—C2—C1	60.3 (4)	C8—C9—C10—C11	130.8 (5)
O1—S1—C2—C3	−120.6 (4)	C8—C9—C10—C14	−47.9 (7)
O2—S1—C2—C1	−61.5 (4)	C9—C5—C6—N3	−2.0 (7)
O2—S1—C2—C3	117.7 (4)	C9—C10—C11—C12	−178.5 (5)
C1—C2—C3—S2	179.2 (3)	C9—C10—C14—C13	178.4 (5)
C1—C2—C3—C4	−1.5 (8)	C10—C11—C12—N4	0.1 (9)
N3—C7—C8—C9	−2.4 (8)	C11—C10—C14—C13	−0.3 (8)
N4—C13—C14—C10	0.2 (8)	C12—N4—C13—C14	0.1 (8)
C5—C9—C10—C11	−48.7 (7)	C13—N4—C12—C11	−0.2 (8)
C5—C9—C10—C14	132.6 (5)	C14—C10—C11—C12	0.2 (8)

Symmetry code: (i) −*x*+1, *y*, −*z*+3/2.

### Bis(4,4'-bipyridin-1-ium) cis-bis(1,2-dicyano-2-sulfidoethene-1-sulfinato-κ2S,S')platinate(2-) Hydrogen-bond geometry (Å, º)

**Table:**

*D*—H···*A*	*D*—H	H···*A*	*D*···*A*	*D*—H···*A*
N4—H4···N3^ii^	0.86	1.88	2.730 (5)	171
C7—H7···N2^iii^	0.93	2.50	3.363 (7)	155
C7—H7···O2^i^	0.93	2.57	3.197 (7)	125
C8—H8···O1	0.93	2.40	3.301 (6)	164
C8—H8···O2^i^	0.93	2.77	3.287 (7)	116

Symmetry codes: (i) −*x*+1, *y*, −*z*+3/2; (ii) *x*, −*y*, *z*−1/2; (iii) *x*, −*y*+1, *z*+1/2.
